# Examining Mental Health Changes Before and After Rhinoplasty: An Analytical Study

**DOI:** 10.1002/pchj.820

**Published:** 2024-12-22

**Authors:** Ramyar Farzan, Afrooz Haghdoost, Mohammad Tolouie, Sakineh Pourgholami Koudehi, Elham Ebrahimi Khonacha, Paria Nikinia, Mojdeh Esmailzadeh

**Affiliations:** ^1^ Department of Plastic & Reconstructive Surgery School of Medicine, Guilan University of Medical Sciences Rasht Iran; ^2^ Department of General Surgery School of Medicine, Guilan University of Medical Sciences Rasht Iran; ^3^ Burn and Regenerative Medicine Research Center Guilan University of Medical Sciences Rasht Iran; ^4^ School of Medicine Guilan University of Medical Sciences Rasht Iran

**Keywords:** Iran, mental health, plastic surgery, Rhinoplasty

## Abstract

This study aimed to explore the mental health status of individuals seeking rhinoplasty before and 3 months after surgery. This study was conducted in 2022 at Velayat Hospital, involving patients aged 18–60 who were seeking rhinoplasty, selected using convenience sampling method. Exclusion criteria included prior nasal surgery and severe psychiatric disorders without clearance from a psychiatrist. The Symptom Check List‐90‐Revised questionnaire was employed to assess psychological disorders across 9 dimensions. Sixty patients were examined, consisting of 14 men and 46 women, with an average age of 31 years. A substantial 83.3% of participants sought rhinoplasty solely for cosmetic reasons, whereas 16.7% cited both cosmetic and breathing issues as motivations. Initially, 59 patients had scores indicating “good” mental health, with one showing “moderate” mental health. Three months after the surgery, all patients scored within “good” mental health. The findings revealed significant improvements in various dimensions of mental health, including depression, anxiety, and obsessive compulsive symptoms, with the exception of paranoid ideation. All three primary indicators of mental health demonstrated significant decrease post‐surgery. The study highlights the positive impact of rhinoplasty on the mental health of patients, particularly among women, individuals over 20, and non‐smokers. Notable improvements were observed across various mental health dimensions, with significant enhancements reported three months post‐surgery. However, the findings underscore the need for careful consideration of psychological conditions when selecting candidates for surgery. Limitations, including a small male sample and lack of a control group, suggest further research is necessary.

## 1 Introduction

Rhinoplasty is a complex cosmetic surgery performed to enhance the appearance of the nose and is one of the most common cosmetic procedures. Rhinoplasty is the third most common cosmetic surgery in the United States and is also the most frequently performed cosmetic surgery in Iran, accounting for about 60% of all cosmetic surgeries conducted in the country (Ebrahimi et al. 2016; Kalantar Hormozi et al. 2018).

In recent years, researchers paid considerable attention to psychiatric issues in patients seeking cosmetic surgery, and pre‐operative psychiatric evaluations have yielded significant findings. Some studies suggest that individuals seeking cosmetic surgery may suffer from various mental health disorders or at least exhibit psychological symptoms or diagnoses based on DSM criteria. Other research indicates that these patients' psychological issues and social concerns might contribute to their desire for cosmetic surgery, particularly for facial and nasal procedures (AlAwadh et al. 2024; Amodeo 2007; Nabavizadeh et al. 2023).

In this context, the question arises whether cosmetic surgery affects patients' mental health. Some studies suggest that rhinoplasty may improve an individual's appearance and lead to better mental health. Additionally, several studies have identified cosmetic surgeries as factors contributing to psychological changes, including increased self‐confidence and a more positive self‐image. They indicate that successful rhinoplasty can generally enhance health‐related quality of life by increasing self‐confidence and reducing anxiety symptoms (Borujeni et al. 2020; Brucoli et al. 2019; Chen and Zhou 2023).

It seems that the desire to undergo cosmetic surgery is influenced by individual, social, and psychological factors. Despite the high rate of rhinoplasty procedures in Iran, there are few studies that have examined patients' mental health, the factors influencing it, and the impact of cosmetic surgery on patients' mental well‐being. Additionally, there is a lack of extensive psychological research on rhinoplasty candidates in Iran. Assessing underlying psychological disorders in patients who are candidates for rhinoplasty is crucial in selecting suitable patients and plays a role in preventing complications and dissatisfaction with the surgery. It could also provide a foundation for psychological studies and complementary treatments for this group of patients (Borujeni et al. 2020).

Therefore, in this study, we aimed to assess the mental health status of patients seeking rhinoplasty at the Velayat Research and Medical Center. Additionally, we intend to evaluate their mental health status within a specified period after the surgery.

## 2 Methods

This study was conducted in 2022 at Velayat Hospital. The study population consisted of patients who were candidates for rhinoplasty in the plastic surgery subspecialty department, with sampling conducted using a convenience method. The study tools included a researcher‐designed form for recording demographic and clinical information, as well as the Symptom Check List‐90‐Revised (SCL‐90‐R) questionnaire, which was used for data collection.

The inclusion criteria for the study were patients seeking rhinoplasty between the ages of 18 and 60. The exclusion criteria were a history of nasal surgery (either cosmetic or functional) and patients under 18 or over 60 years of age. It is important to note that patients with severe psychiatric disorders (as documented in their medical records), such as major depressive disorder, severe obsessive compulsive disorder, and others, who had not recently been under the care of their psychiatrist and had not received psychiatric clearance for surgery, were not considered suitable candidates for rhinoplasty based on the patient selection policies of the plastic surgery department at Velayat Hospital. Therefore, these patients were not included in the study. These patients were first referred to their psychiatrist and after their severe symptoms were managed and they received approval from psychiatrist could they be considered candidates for cosmetic surgery.

In this study, the SCL‐90‐R questionnaire was used to assess psychological disorders. The SCL‐90‐R questionnaire includes 90 questions across 9 different dimensions: somatization, obsessive compulsive, interpersonal sensibility, depression, anxiety, anger hostility, phobic anxiety, paranoid ideation, and psychoticism. The interpretation of the questionnaire is based on three indices: Global Severity Index (GSI), Positive Symptom Distress Index (PSDI) and Positive Symptom Total (PST). This questionnaire has been utilized in various epidemiological studies, and in a study conducted by Modabernia et al. (2010) in Iran, the test–retest reliability was reported as 0.93, and the reliability of the questionnaire based on Cronbach's alpha was reported as 0.85 (Akhavan Abiri and Shairi 2020; Kass et al. 1983).

The objectives and procedures of the research were explained to the patients. The patients were also assured that their information would remain confidential. All participants were fully informed of the study and informed consent was obtained from all patients. The study was approved by the Ethics Committee of Research and Technology Deputy of Guilan University of Medical Sciences.

After obtaining informed consent, the patients were enrolled in the study. The interviewers (who were master's level psychologists) conducted interviews with each patient both before rhinoplasty and three months afterward. During a session lasting approximately one hour, they completed the initial form and the SCL‐90‐R questionnaire for each patient.

In the data analysis for the study, both descriptive and inferential methods were employed. The descriptive analysis utilized measures of central tendency, while the inferential analysis used the Kolmogorov–Smirnov test to assess the normality of the quantitative variables. For variables that followed a normal distribution, a paired *t*‐test was used to compare two dependent groups. For variables that did not follow a normal distribution, the Wilcoxon or McNemar tests were applied. Data analysis was conducted using SPSS20 statistical software, with a significance level set at less than 0.05.

## 3 Results

In this study, 60 patients were examined, of whom 14 were men and 46 were women. The average age of the patients seeking rhinoplasty was 31 years. The youngest patient was 18 years old, and the oldest was 54 years old. Among the participants, 28.3% were employed, and all individuals were literate. Additionally, 65% of the patients were married. A total of 83.3% of the study population cited cosmetic reasons as their sole motivation for seeking rhinoplasty, while 16.7% mentioned both cosmetic reasons and breathing issues as their motivation for undergoing the surgery. Other descriptive findings are reported in Table 1.

**TABLE 1:** Individual characteristics and descriptive findings of rhinoplasty candidate patients in the study.

Variable	Condition	Number	Percent (%)
Gender	Male	14	23.3
	Female	46	76.7
Age (year)	Less than 20 years old	10	16.7
	21–30 year old	18	30
	31–40 year old	21	35
	More than 40 year old	11	18.3
Age (year) Mean ± SD(min—max)		31.88 ± 9.54 (18–54)	
Employment status	Employed	23	38.3
	Unemployed—housewife	32	53.3
	Student	5	8.3
Education	Pre‐university	32	53.3
	College education	28	46.7
Marital status	Married	39	65
	Single	20	33.3
	Divorced	1	1.7
Smoking	Yes	9	15
	No	51	85
Alcohol consumption	Yes	7	11.7
	No	53	88.3
The cause of rhinoplasty	Cosmetics	50	83.3
	Breathing disorder	0	0
	Cosmetics and breathing disorder	10	16.7

Table 2 presents the overall mental health status of rhinoplasty candidates using the SCL‐90‐R questionnaire before and three months after the operation. Initially, 59 patients scored between 0 and 120 (good mental health), while one patient scored between 121 and 240 (moderate mental health). In the postoperative reassessment using the SCL‐90‐R questionnaire, all 60 patients scored between 0 and 120 (good mental health).

**TABLE 2:** Mental health status in rhinoplasty candidates based on SCL‐90‐R questionnaire before and three months after surgery.

	Before rhinoplasty		3 months after rhinoplasty	
Mental health status	Number	Percent (%)	Number	Percent (%)
Good (0–120)	59	98.3	60	100
Moderate (121–240)	1	1.7	0	0
Low (241–360)	0	0	0	0
Total	60	100	60	100

Table 3 displays the average scores across the nine dimensions of mental health in the SCL‐90‐R questionnaire. Before the operation, the highest scores were recorded in the areas of depression, interpersonal sensibility, paranoid ideation, obsessive compulsive, anger hostility, anxiety, somatization, psychoticism, and phobic anxiety. Three months after surgery, there was a statistically significant decrease in average scores across all but the paranoid dimension. Additionally, the three main indicators of the questionnaire, including GSI, PST, and PSDI, exhibited a significant decrease in scores.

**TABLE 3:** Comparison of patient's scores in different dimensions of the SCL‐90‐R questionnaire before and three months after rhinoplasty.

Mental health dimensions	SCL‐90‐R Scores before rhinoplasty	SCL‐90‐R Scores 3 months after rhinoplasty	*p*
Somatization	4.08 ± 4.78	1.88 ± 2.52	0.004
Obsessive compulsive	5.36 ± 0.69	3.22 ± 0.41	0.000
Interpersonal sensibility	7.61 ± 4.86	4.1 ± 2.55	0.000
Depression	7.68 ± 7.49	5.63 ± 3.76	0.002
Anxiety	4.15 ± 4.82	1.9 ± 2.13	< 0.001
Anger hostility	4.68 ± 4.09	3.58 ± 2.3	0.013
Phobic‐anxiety	2.01 ± 3.4	0.63 ± 0.95	0.002
Paranoid—ideation	7.38 ± 4.29	7.43 ± 2.8	0.908
Psychoticism	3.55 ± 3.44	1.18 ± 1.77	< 0.001
Additional items	2.78 ± 0.35	1.8 ± 0.23	< 0.001
Total SCL‐90‐R Score	35.79 ± 4.62	34.3 ± 16.45	< 0.001
GSI Score	0.39 ± 0.05	0.38 ± 0.02	< 0.001
PST Score	30.95 ± 16.17	22.25 ± 9.34	< 0.001
PSDI Score	1.62 ± 0.36	1.5 ± 0.22	0.011

*Note: p*‐values highlighted in gray were significant.

Tables 4, 5, 6 illustrate the scores obtained in GSI, PSDI, and PST separately, considering various variables. Table 4, for instance, reveals that in men, there was no statistically significant difference in GSI values before and 3 months after rhinoplasty surgery (*p* = 0.074). Conversely, a significant difference was observed in women (*p* < 0.001). Table 4 indicates that there was no statistically significant difference in GSI values before and 3 months after rhinoplasty in individuals below the age of 20 (*p* = 0.194). However, a statistically significant difference was observed in patients aged 20 and above. Regarding job status, Table 4 shows no statistically significant difference between GSI values before and 3 months after rhinoplasty surgery in students (*p* = 0.271). In contrast, a significant difference was noted among working individuals or housewives. Furthermore, the study's data revealed a statistically significant difference between GSI values before and 3 months after rhinoplasty surgery across all educational groups and among individuals with diverse marital statuses.

**TABLE 4:** Comparison of Global Severity Index scores before and three months after rhinoplasty surgery based on the variables examined in patients.

Variable	Condition	GSI Scores before rhinoplasty	GSI Scores 3 months after rhinoplasty	*p*
Gender	Male	0.45 ± 0.3	0.31 ± 0.15	0.074
	Female	0.63 ± 0.41	0.4 ± 0.18	< 0.001
Age (year)	Less Than 20 Years Old	0.41 ± 0.26	0.34 ± 0.13	0.194
	21–30 years old	0.54 ± 0.36	0.31 ± 0.17	0.000
	31–40 years old	0.64 ± 0.47	0.43 ± 0.21	0.011
	More than 40 years old	0.74 ± 0.36	0.41 ± 0.13	0.009
Employment status	Employed	0.54 ± 0.34	0.34 ± 0.17	0.002
	Unemployed—housewife	0.66 ± 0.44	0.41 ± 0.19	< 0.001
	Student	0.39 ± 0.17	0.31 ± 0.09	0.171
Education	Pre‐university	0.5 ± 0.3	0.37 ± 0.15	< 0.001
	College education	0.69 ± 0.49	0.39 ± 0.2	< 0.001
Marital status	Married	0.64 ± 0.44	0.38 ± 0.19	< 0.001
	Single‐divorced	0.49 ± 0.28	0.36 ± 0.15	0.004
Smoking	Yes	0.56 ± 0.32	0.34 ± 0.11	0.077
	No	0.59 ± 0.41	0.38 ± 0.19	< 0.001
Alcohol consumption	Yes	0.53 ± 0.27	0.37 ± 0.14	0.095
	No	0.6 ± 0.41	0.38 ± 0.18	< 0.001
The cause of rhinoplasty	Cosmetics	0.61 ± 0.4	0.38 ± 0.18	< 0.001
	Cosmetics and breathing disorder	0.5 ± 0.36	0.36 ± 0.19	0.14

*Note: p*‐values highlighted in gray were significant.

**TABLE 5:** Comparison of Positive Symptom Total scores before and three months after rhinoplasty surgery based on the investigated variables in patients.

Variable	Condition	PST Score before rhinoplasty	PST Score 3 months after rhinoplasty	*p*
Gender	Male	26.28 ± 12.64	17.85 ± 9.04	0.017
	Female	32.36 ± 16.96	23.58 ± 9.11	< 0.001
Age (year)	Less than 20 years old	24.1 ± 12.04	20.1 ± 7.38	0.071
	21–30 years old	28.94 ± 14.76	19.27 ± 8.66	< 0.001
	31–40 years old	23.76 ± 18.23	24.95 ± 11.16	0.005
	More than 40 years old	35.09 ± 16.93	23.9 ± 7.07	0.044
Employment status	Employed	30.43 ± 15.93	19.78 ± 8.94	< 0.001
	Unemployed—housewife	32.25 ± 17.08	24.34 ± 9.66	< 0.001
	Student	25 ± 11.7	20.2 ± 6.97	0.176
Education	Pre‐university	26.81 ± 12.39	21.65 ± 8.23	0.008
	College education	35.67 ± 18.74	22.92 ± 10.58	< 0.001
Marital status	Married	32.35 ± 17.19	22.76 ± 10.34	< 0.001
	Single‐divorced	20.33 ± 14.09	21.28 ± 7.25	0.004
Smoking	Yes	31 ± 12.81	19.11 ± 6.71	0.028
	No	30.94 ± 16.8	22.8 ± 9.68	< 0.001
Alcohol consumption	Yes	29.71 ± 9.28	20.71 ± 7.63	0.034
	No	31.11 ± 16.92	22.45 ± 9.59	< 0.001
The cause of rhinoplasty	Cosmetics	31.74 ± 16.19	22.4 ± 9.22	< 0.001
	Cosmetics and breathing disorder	27 ± 16.3	21.5 ± 10.42	0.217

*Note: p*‐values highlighted in gray were significant.

**TABLE 6:** Comparison of Positive Symptom Distress Index values before and three months after rhinoplasty surgery based on the variables investigated in patients.

Variable	Condition	PSDI Score before rhinoplasty	PSDI score 3 months after rhinoplasty	*p*
Gender	Male	1.45 ± 0.27	1.57 ± 0.21	0.177
	Female	1.67 ± 0.37	1.48 ± 0.22	< 0.001
Age (year)	Less than 20 years old	1.5 ± 0.31	1.52 ± 0.23	0.859
	21–30 years old	1.56 ± 0.39	1.41 ± 0.23	0.07
	31–40 years old	1.59 ± 0.36	1.55 ± 0.24	0.697
	More than 40 years old	1.89 ± 0.22	1.53 ± 0.06	< 0.001
Employment status	Employed	1.5 ± 0.31	1.51 ± 0.24	0.941
	Unemployed—housewife	1.74 ± 0.38	1.51 ± 0.2	< 0.001
	Student	1.41 ± 0.13	1.44 ± 1.18	0.835
Education	Pre‐university	1.63 ± 0.34	1.51 ± 0.18	0.067
	College education	1.61 ± 0.39	1.49 ± 0.26	0.085
Marital status	Married	1.69 ± 0.37	1.5 ± 0.21	< 0.001
	Single‐divorced	1.49 ± 0.32	1.51 ± 0.23	0.703
Smoking	Yes	1.56 ± 0.27	1.63 ± 0.14	0.59
	No	1.63 ± 0.37	1.48 ± 0.22	0.002
Alcohol consumption	Yes	1.54 ± 0.36	1.64 ± 0.16	0.541
	No	1.63 ± 0.36	1.49 ± 0.22	0.003
The cause of rhinoplasty	Cosmetics	1.63 ± 0.37	1.5 ± 0.23	0.012
	Cosmetics and breathing disorder	1.59 ± 0.34	1.52 ± 0.17	0.55

*Note: p*‐values highlighted in gray were significant.

In terms of smoking and alcohol consumption, the paired *t*‐test revealed no statistically significant difference between GSI values before and 3 months after rhinoplasty surgery in smokers (*p* = 0.077). However, in non‐smokers, this difference was significant (*p* < 0.001). Similar findings were observed concerning alcohol consumption.

A statistically significant difference was noted between GSI values before and 3 months after rhinoplasty surgery in individuals whose reason for referral was cosmetic surgery (*p* < 0.001). Contrastingly, this difference was not significant in individuals with cosmetic‐breathing problems as the motivating factor (*p* = 0.14).

Table 5 shows that the PST score significantly decreased in many variables three months after rhinoplasty. The PST scores of both men and women in the study experienced a significant decrease three months post‐surgery (*p* = 0.017 and *p* < 0.001). Across all age groups (except for those under 20) and educational levels, the PST score showed a statistically significant decrease three months after rhinoplasty. Among those who underwent rhinoplasty for cosmetic reasons, the PST score significantly decreased (*p* < 0.001). However, in patients who had the surgery for both cosmetic and breathing issues, while the PST score decreased, it was not statistically significant (*p* = 0.217).

Table 6 also demonstrates a decrease in PSDI scores three months after rhinoplasty across most variables. However, this decrease was only statistically significant in some groups, such as women, housewives, married individuals, and non‐smokers and non‐alcoholics. Additionally, the decrease in PSDI scores was significant in patients who had rhinoplasty for cosmetic purposes (*p* = 0.012). However, similar to the PST score, the decrease in PSDI scores in patients with both cosmetic and breathing issues was not statistically significant (*p* = 0.55).

Table 7 displays the mean score for each dimension of mental health before and after surgery, categorized by gender. In the male participants, the highest scores were observed in the following order: Obsessive Compulsive, Paranoid Ideation, Interpersonal Sensibility, Depression, Anger Hostility, Psychoticism, Anxiety, Somatization, and finally Phobic Anxiety. A significant decrease in scores was noted post‐surgery in the areas of Obsessive Compulsive, Interpersonal Sensitivity, and Psychoticism, while in other dimensions, the decrease in scores was not statistically significant.

**TABLE 7:** Comparison of the average scores of different dimensions of mental health before and after surgery according to gender.

Mental health dimensions	Male			Female		
	Average score before Rhinoplasty	Average score 3 months after Rhinoplasty	*p*‐value	Average score before Rhinoplasty	Average score 3 months after Rhinoplasty	*p*‐value
Somatization	1.57 ± 2.53	0.64 ± 0.74	0.543	4.84 ± 5.05	2.26 ± 2.75	0.004
Obsessive compulsive	7.78 ± 4.75	5.42 ± 3.08	0.000	8.91 ± 5.55	6.1 ± 3.28	0.09
Interpersonal sensibility	6.5 ± 4.32	3.71 ± 2.72	0.024	7.95 ± 5	4.21 ± 2.52	< 0.001
Depression	4 ± 4.52	2.54 ± 2.4	0.256	8.8 ± 7.88	6.54 ± 3.64	0.005
Anxiety	2.35 ± 2.84	1.64 ± 1.54	0.437	4.69 ± 5.18	1.97 ± 2.29	< 0.001
Anger hostility	4 ± 3.01	3.85 ± 2.85	0.818	4.89 ± 4.37	3.5 ± 2.14	0.011
Phobic‐ anxiety	1.14 ± 1.29	0.57 ± 0.85	0.13	2.28 ± 3.79	0.65 ± 0.99	< 0.001
Paranoid ‐ideation	7.57 ± 3.3.61	6.85 ± 2.5	0.377	7.32 ± 4.51	7.6 ± 2.88	0.582
Psychoticism	2.92 ± 3.7	0.92 ± 1.2	0.036	3.73 ± 3.38	1.26 ± 1.91	< 0.001
Additional items	2.78 ± 2.57	2.07 ± 1.81	0.253	3.84 ± 2.82	1.97 ± 1.81	< 0.001

*Note: p*‐values highlighted in gray were significant.

In the female participants, the highest scores were observed in the following order: Obsessive Compulsive, Depression, Interpersonal Sensitivity, Paranoid Ideation, Anger Hostility, Somatization, Anxiety, Psychoticism, and finally Phobic Anxiety. A statistically significant decrease in scores after rhinoplasty was observed in all dimensions except for Paranoid Ideation (*p* = 0.582).

## 4 Discussion

The present study aimed to examine the mental health of patients undergoing rhinoplasty before and after the procedure. Most of the patients in this study were married women aged 20 to 40. Similar to this study, in most research within the field of plastic and cosmetic surgery, the majority of applicants are young women, while men consistently represent a smaller percentage of cosmetic surgery candidates (AlAwadh et al. 2024; Nabavizadeh et al. 2023; Shauly et al. 2020).

The most significant finding of the present study was the statistically significant decrease in scores after surgery. A meaningful improvement in the mental health status of patients was observed across many dimensions three months post‐rhinoplasty. Based on the average scores obtained in various dimensions of mental health among all patients, the highest scores were seen in the following order: depression, interpersonal sensibility, paranoid ideation, obsessive compulsive, anger hostility, anxiety, somatization, psychoticism, and phobic anxiety. After rhinoplasty, a significant improvement was noted in all these areas except for paranoid ideation.

These results were also noteworthy when analyzed by gender among the patients. In the male participants, the highest scores were found in the dimensions of Obsessive Compulsive, Paranoid Ideation, and Interpersonal Sensibility. In women, with slight differences, the highest scores were observed in the following order: Obsessive Compulsive, Depression, and Interpersonal Sensibility. In this study, a significant decrease in scores after rhinoplasty was noted in women across all dimensions except for Paranoid Ideation. However, in men, this decrease was not statistically significant in most dimensions. It appears that female patients benefited more from the surgery in terms of improving their mental health status.

Studies have shown that successful rhinoplasty can improve quality of life by enhancing mental health, increasing self‐confidence, and reducing symptoms of anxiety (Alanko et al. 2017; Hashemi et al. 2020; Tasman 2010). In a similar study conducted by Ghahramani and colleagues in 2018, the average age of the patients studied was 29 ± 8 years, with the majority being women. In their statistical population, the highest scores were related to Depression, Obsessive Compulsive, and Somatization. Their study indicated a significant decrease in patients' scores after rhinoplasty (Ghahremani et al. 2019). In another similar study, different results were obtained. Baghan and colleagues reported in Iran in 2015 that although cosmetic surgery did not lead to an increase in symptoms of psychological disorders among patients, it also did not contribute to the improvement of their mental health (Baghban Boosari, Nemati, and Rezaei 2016). Given the differing results of these studies, further investigation into the mental health of patients undergoing cosmetic surgery and the effects of cosmetic procedures on their mental well‐being is warranted.

The findings of this study indicated the effect of some variables on the improvement of mental health scores after surgery. The findings showed that mental health scores were significantly better in women, patients over the age of 20, and non‐smokers and non‐alcoholics, compared to men, those under 20, and smokers and alcoholics, across various dimensions of mental health. Additionally, patients who underwent rhinoplasty for cosmetic reasons showed a significant improvement in mental health indicators compared to those who underwent rhinoplasty for both breathing and cosmetic issues. On the other hand, marital status and education level did not affect patients' mental health scores after surgery, as patients with varying marital statuses and educational backgrounds experienced a significant improvement in their mental health indicators.

The findings of this study suggest that rhinoplasty may improve mental health in individuals who appear psychologically healthy and have no history of severe psychiatric illness. Postoperative scores showed significant improvement in our patients who had no history of major psychiatric disorders upon entering the study. However, a key question arises: does this improvement also occur in individuals with poor mental health or more severe psychological disorders? Some studies have indicated that certain mental health issues (such as anxiety and depression) may be the primary motivation for seeking cosmetic rhinoplasty, but these same issues may hinder positive outcomes. Research also suggests that psychological outcomes in patients with major depressive disorder, severe personality disorders, and psychosis are often unsatisfactory, with complaints generally not improving and, in some cases, even worsening (Alanko et al. 2017; Amodeo 2007; Shauly et al. 2020; Tasman 2010; Tavassoli et al. 2022). However, there is a consensus that psychiatric disorders or psychological distress, except in severe cases, are not necessarily a contraindication for surgery, especially when accompanied by support and an understanding of the patient's motivations (Sun and Rieder 2022).

The limitations of this study include the small number of male patients and the limited number of patients with moderate and poor mental health scores, as well as the lack of a control group, which necessitates caution in generalizing the results. To address these limitations, future studies could benefit from a larger and more diverse statistical population, incorporating participants from multiple centers to enhance the external validity of the findings. Controlled studies with well‐matched control groups would provide a more strong foundation for drawing conclusions about the specific impact of rhinoplasty on mental health and it is recommended that future studies conduct a multivariable meta‐regression analysis to examine possible independent predictors of improvement in mental health. Moreover, future investigations should deliberately focus on individuals with lower initial mental health scores to comprehensively assess the potential benefits and risks associated with rhinoplasty in this subgroup.

In summary, while our study offers valuable insights into the relationship between rhinoplasty and mental health, the identified limitations underscore the necessity for continued research endeavors with more comprehensive methodologies. Addressing these limitations will contribute to a more nuanced understanding of the complex interplay between cosmetic surgery interventions and mental health outcomes.

## 5 Conclusion

This study underscores the positive impact of rhinoplasty on mental health indicators, with notable improvements observed, particularly among women, individuals over the age of 20, and those who do not smoke or consume alcohol. However, the findings emphasize the importance of considering the psychological conditions and mental health of patients when selecting suitable candidates for rhinoplasty. Informed decision‐making in this regard can contribute to optimizing outcomes and patient satisfaction.

Furthermore, it is recommended that plastic surgeons integrate pre‐operative psychiatric counseling, especially for patients exhibiting signs of mental health disorders. This approach can enhance the overall care and well‐being of patients undergoing rhinoplasty, ensuring a comprehensive consideration of both physical and psychological aspects throughout the surgical process.

## Conflicts of Interest

The authors declare no conflicts of interest.

## References

[pchj820-bib-0001] Akhavan Abiri, F. , and M. R. Shairi . 2020. “Validity and Reliability of Symptom Checklist‐90‐Revised (SCL‐90‐R) and Brief Symptom Inventory‐53 (BSI‐53).” Clinical Psychology and Personality 17, no. 2: 169–195. 10.22070/cpap.2020.2916.

[pchj820-bib-0002] Alanko, O. , M. T. Tuomisto , T. Peltomäki , M. Tolvanen , T. Soukka , and A.‐L. Svedström‐Oristo . 2017. “A Longitudinal Study of Changes in Psychosocial Well‐Being During Orthognathic Treatment.” International Journal of Oral and Maxillofacial Surgery 46, no. 11: 1380–1386. 10.1016/j.ijom.2017.05.004. 28579266

[pchj820-bib-0003] AlAwadh, I. , A. Bogari , T. Azhar , et al. 2024. “Prevalence of Body Dysmorphic Disorder Among Rhinoplasty Candidates: A Systematic Review.” Ear, Nose & Throat Journal 103, no. 6: 377–383. 10.1177/01455613211056543. 34789021

[pchj820-bib-0004] Amodeo, C. A. 2007. “The Central Role of the Nose in the Face and the Psyche: Review of the Nose and the Psyche.” Aesthetic Plastic Surgery 31: 406–410. 10.1007/s00266-006-0241-2. 17551776

[pchj820-bib-0005] Baghban Boosari, M. M. S. , S. H. Nemati , and S. Rezaei . 2016. “Mental Health Status Before and After Cosmetic Rhinoplasty.” Journal of Gorgan University of Medical Sciences 18, no. 2: 84–90.

[pchj820-bib-0006] Borujeni, L. A. , S. Pourmotabed , Z. Abdoli , et al. 2020. “A Comparative Analysis of patients' Quality of Life, Body Image and Self‐Confidence Before and After Aesthetic Rhinoplasty Surgery.” Aesthetic Plastic Surgery 44: 483–490. 10.1007/s00266-019-01559-3. 31832733

[pchj820-bib-0007] Brucoli, M. , R. R. Y. Baena , P. Boffano , and A. Benech . 2019. “Psychological Profiles in Patients Undergoing Orthognathic Surgery or Rhinoplasty: A Preoperative and Preliminary Comparison.” Oral and Maxillofacial Surgery 23: 179–186. 10.1007/s10006-019-00758-1. 31016403

[pchj820-bib-0008] Chen, K. , and L. Zhou . 2023. “The Effect of Functional Rhinoplasty on Quality of Life: A Systematic Review and Meta‐Analysis.” Aesthetic Plastic Surgery 1‐8: 847–854. 10.1007/s00266-023-03390-3. 37173413

[pchj820-bib-0009] Ebrahimi, A. , M. H. Kalantar Motamedi , A. Shams , and N. Nejadsarvari . 2016. “Health and Social Problems of Rhinoplasty in Iran.” World Journal of Plastic Surgery 5, no. 1: 75–76. 27308246 PMC4904144

[pchj820-bib-0010] Ghahremani, L. , M. Motevasel , M. Fararooei , and T. Rakhshani . 2019. “Mental Health in Rhinoplasty Applicants, Before and After Surgery.” Archives of Psychiatry and Psychotherapy 4, no. 1: 45–51. 10.12740/APP/106077.

[pchj820-bib-0011] Hashemi, M. , N. Sakhi , H. Ghazavi , P. Bolourinejad , and G. Kheirabadi . 2020. “Effects of Aesthetic Rhinoplasty on Quality of Life, Anxiety/Depression, and Self‐Esteem of the Patients.” European Journal of Plastic Surgery 43: 153–158. 10.1007/s00238-019-01582-2.

[pchj820-bib-0012] Kalantar Hormozi, A. , S. Maleki , A. Rahimi , A. Manafi , and S. J. Amirizad . 2018. “Cosmetic Surgery in Iran: Sociodemographic Characteristics of Cosmetic Surgery Patients in a Large Clinical Sample in Tehran.” American Journal of Cosmetic Surgery 35, no. 4: 177–182. 10.1177/0748806818764734.

[pchj820-bib-0013] Kass, F. , E. Charles , D. F. Klein , and P. Cohen . 1983. “Misuse of the Symptom Checklist 90‐Reply.” Archives of General Psychiatry 40, no. 10: 1152–1153. 10.1001/archpsyc.1983.01790090114026. 6625866

[pchj820-bib-0014] Modabernia, M. , H. S. Tehranie , M. Falahi , and M. Faghirpour . 2010. “Normalizing SCL‐90‐R Inventory in Guilan High‐School Students.” Journal of the Guilan University of Medical Sciences 19, no. 75: 58–65.

[pchj820-bib-0015] Nabavizadeh, S. S. , R. Naseri , E. Sadeghi , A. Afshari , N. Dehdari Ebrahimi , and A. Sadeghi . 2023. “Prevalence of Body Dysmorphic Disorder in Rhinoplasty Candidates: A Systematic Review and Meta‐Analysis.” Health Science Reports 6, no. 8: e1495. 10.1002/hsr2.1495. 37599660 PMC10435832

[pchj820-bib-0016] Shauly, O. , J. Calvert , G. Stevens , R. Rohrich , N. Villanueva , and D. J. Gould . 2020. “Assessment of Wellbeing and Anxiety‐Related Disorders in Those Seeking Rhinoplasty: A Crowdsourcing‐Based Study.” Plastic and Reconstructive Surgery Global Open 8, no. 4: e2737. 10.1097/GOX.0000000000002737. 32440408 PMC7209878

[pchj820-bib-0017] Sun, M. D. , and E. A. Rieder . 2022. “Psychosocial Issues and Body Dysmorphic Disorder in Aesthetics: Review and Debate.” Clinics in Dermatology 40, no. 1: 4–10. 10.1016/j.clindermatol.2021.08.008. 35190063

[pchj820-bib-0018] Tasman, A.‐J. 2010. “The Psychological Aspects of Rhinoplasty.” Current Opinion in Otolaryngology & Head and Neck Surgery 18, no. 4: 290–294. 10.1097/MOO.0b013e32833b51e6. 20543695

[pchj820-bib-0019] Tavassoli, A. , M. Zavarmosavi , A. Masoodi , and Z. Geraili . 2022. “Mental Health Status Among the Candidates for Rhinoplasty: A Case‐Control Study.” Journal of Current Oncology and Medical Sciences 2, no. 3: 257–265.

